# Assessing the effect of Gotfried reduction with positive buttress pattern in the young femoral neck fracture

**DOI:** 10.1186/s13018-020-02039-0

**Published:** 2020-11-07

**Authors:** Kai Huang, Xiaohui Fang, Guijun Li, Jiajun Yue

**Affiliations:** 1 Department of Orthopedics, Changzhou Cancer Hospital Soochow University, No. 68 Honghe Road, Changzhou, 213001 Jiangsu China; 2 Department of Intensive Care Unit, Changzhou Cancer Hospital Soochow University, No. 68 Honghe Road, Changzhou, 213001 Jiangsu China

**Keywords:** Femoral neck fracture, Internal fixation, Gotfried reduction, Positive buttress, Negative buttress

## Abstract

**Background:**

Although many available surgical procedures for displaced femoral neck fractures in young patients, there are still many challenges to achieve satisfactory results. The incidence of avascular necrosis and nonunion rates remains relatively high despite the progress in our understanding and surgical technique. The purpose of this study was to evaluate the clinical efficacy of Gotfried reduction and cannulated screw fixation in the treatment of femoral neck fracture for young adults.

**Methods:**

A retrospective analysis was made on 67 cases from May 2013 to March 2019. They were divided into three groups according to the first postoperative anteroposterior view of hip X-ray: Anatomic reduction (group A), Gotfried positive buttress reduction (group B), and Gotfried negative buttress reduction (group C). The incidence of avascular osteonecrosis of the femoral head (AVN) and the Harris scores of hip joints were compared in three groups at the last follow-up.

**Results:**

The mean follow-up period after surgery was 22.5 ± 11.3 (range, 11–34) months. There were 21 cases (mean age, 49.7 ± 11.6) in group A, 24 cases (mean age, 48.6 ± 11.3) in group B, 22 cases (mean age, 48.3 ± 12.4) in group C. No significant difference in general preoperative demographics (*P* > 0.05). The incidence of avascular necrosis of femoral head in group A, B, and C was 19.05%, 20.83%, and 22.73%, respectively, showing no significant difference between groups (*P* = 0.156). The mean Harris hip scores at the final follow-up for groups A (85.6 ± 6.7) and B (84.5 ± 6.2) were significantly higher than group C (74.3 ± 8.3), and the difference was statistically significant (*P* = 0.043). The incidence of femoral neck shortening in group A and group B was significantly lower than that in group C in postoperative 1 year, and the difference was statistically significant (*P* < 0.05).

**Conclusions:**

Gotfried positive buttress reduction and fixation for femoral neck fracture may lead to similar clinical results with anatomic reduction, but much better than Gotfried negative buttress reduction. For the patients of femoral neck fracture with severe displacement and difficulty reduction, it is not necessary to pursue anatomical reduction. Achieving positive valgus reduction can also obtain satisfactory clinical results, and should try to avoid negative buttress.

## Introduction

Femoral neck fractures in young patients usually result from high-energy injuries, the treatment remains a challenging issue for orthopedic surgeons. Displaced femoral neck fractures are usually accompanied by higher complications, such as avascular necrosis, fracture nonunion, and femoral neck shortening [1, 2]. These complications are the leading cause of re-operating [1–5]. In elder patients, the preferred treatment for a displaced femoral neck fracture is hemiarthroplasty or total hip replacement (THR) in the majority of cases. However, younger patients with femoral neck fractures are a particular clinical research group. Different from elderly patients, premature hip replacement may increase the risk of revision rate [6–9]. Therefore, reduction of the fracture, rigid fixation until the fracture heals, and retention of the hip joint are the primary treatment goals. Closed reduction and internal fixation with multiple cannulated screws are common methods for treating displaced femoral neck fractures [9–11].

The quality of reduction has been considered to be one of the most significant factors of successful treatment. Meanwhile, anatomic reduction can promote fracture healing and avoid complications. So, there seems to be a consensus that an anatomical reduction is mandatory [4]. However, in 2013, Gotfried et al. proposed the concept of “non-anatomical reduction of unstable subcapital femoral neck fractures” [12]. Even though only 5 case data were provided, the clinical results were satisfactory. However, this may suggest that successful anatomical reduction does not guarantee an uneventful fracture healing and no failure.

## Patients and methods

### Inclusion and exclusion criteria

Inclusion criteria: (1) All patients with age of ≤ 65 years, who were diagnosed with femoral neck fracture based on both imaging and physical examination. (2) The procedure was Gotfried closed reduction and internal fixation with cannulated cancellous screws. (3) Patients had no hip dysplasia neither arthritis before the injury. (4) All cases had at least 1 year of complete follow-up data.

Exclusion criteria: (1) Pathological fractures, acetabular injuries, preexisting ipsilateral hip diseases, or femoral head fracture. (2) Multiple-trauma patients. (3) Traumatic brain injury and Glasgow score < 14. (4) Patients with severe cognitive impairment and mental illness.

Besides, we were using Garden’s index to assess the fracture alignment after the reduction. Generally, those reductions fulfilling the Garden’s criteria (160 to 180° in anteroposterior and lateral plane) were considered acceptable, and those did not were excluded.

The Institutional Ethics Committee approved the present study of our hospital. All patients were included in the study after signing the informed consent.

### The general information

From May 2013 to March 2019, 67 patients finally met the inclusion criteria and had complete follow-up data. They were divided into three groups according to the first imaging results after surgery. Group A was the anatomical reduction group, group B was Gotfried positive buttress reduction group, and group C was Gotfried negative buttress reduction group. There were 21 patients in group A, the average age was 49.7 ± 11.6 years; 24 cases in group B, the average age was 48.6 ± 11.3 years; 22 cases of group C, mean age was 48.3 ± 12.4 years; general information of the patients are shown in Table 1.

**Table 1:** Basic characteristics of patients

Group	Case	Mean age $\Big(\overline{x}\pm s$)	Gender		Classification (Garden)				Follow-up time (month) ($\overline{x}\pm s$)
			Male	Female	I	II	III	IV	
A	21	49.7 ± 11.6	12	9	7	5	4	5	22.4 ± 10.7
B	24	48.6 ± 11.3	14	10	8	6	5	5	22.1 ± 11.2
C	22	48.3 ± 12.4	13	9	4	7	5	6	22.8 ± 11.6

*Group A* anatomical reduction group, *Group B* Gotfried positive buttress reduction group, *Group C* Gotfried negative buttress reduction group

### Surgical procedures

All fractures underwent Gotfried closed reduction and internal fixation with cannulated cancellous screws. The patient was placed on an orthopedic traction bed after anesthesia, and the perineum was protected against the traction site. The lower limb traction was performed on the operative side: lower limb abduction and knee flexion, hip flexion on the contralateral side for convenient fluoroscopy during operation.

Gotfried closed reduction technology could be divided into three different steps as follows: (1) Gradually increased traction is applied in 2 directions: first, lateral—using a towel wrapped around the upper tight and second longitudinal—on the leg utilizing fracture table. (2) Reduction—while under traction in both directions, the lower limb is brought into adduction and internal rotation. Usually, about 45° adduction is required. The third stage, not presented in the drawing: reconstruction—while in adduction and internal rotation, the release of longitudinal and lateral traction.

(3) Percutaneous cannulated screw fixation: Insert a threaded guide needle in front of the femoral neck to determine the anteversion angle under fluoroscopy. Insert the guide needle at the position of the trochanter slightly above the centerline of the femur. The direction is parallel to the first positioning needle, guide-pin is drilled into place along the medial cortex of the femoral neck and head to within 5 mm of subchondral bone; measure the length of each cannulated screw and screw in the corresponding length of the cannulated screw. The C-arm confirms the fracture reduction quality and the screws’ position to prevent the screw from penetrating the hip joint.

### Postoperative management

Intravenous prophylactic antibiotics (cefazolin 2.0 g) were routinely administered 30 min preoperatively. All patients were kept in absolute bed rest for 3 days and mobilized with crutches without weight bearing on the seventh postoperative day on the affected side. The patients received partial weight-bearing exercises with crutches after 8 weeks, and usual weight-bearing activities after 12 weeks. These patients had a positive compression ultrasound for deep-vein thrombosis and received anticoagulant therapy on the next day after the operation.

### Evaluation criterion and observation index

#### Follow-up data

The follow-up duration was measured from the time of operation to the last follow-up date, and the avascular necrosis of the femoral head, mal-union or nonunion of the fracture, femoral neck shorting, and varus, was recorded at the last follow-up. Shortening and varus collapse were independently assessed by two doctors who were blinded to the functional outcome. We took Zlowodzki’s methods to stratified the degree of shortening and varus collapse into three categories: none/mild (within 5 mm/5°), moderate (5 mm to 10 mm/5 to 10°), and severe (> 10 mm/> 10°). Shortening and varus malalignment were measured on all postoperative radiographs by comparison with the other hip [13]. Harris hip scoring (HHS) system was used to measure the hip function. The quality of fracture reduction is divided into anatomical reduction and non-anatomical reduction. The non-anatomical reduction is divided into Gotfried positive support reduction and negative support reduction (Table 2).

**Table 2:** The incidence of femoral head shorting and varus, AVN, and Harris score at last follow-up

Group	Case	Shorting and varus (case, %)			AVN (case, %)	Harris score
		None/mild	Moderate	Severe		
A	21	16 (76.19%)	4 (19.05%)	1 (4.76%)	4 (4/21, 19.05%)	85.6 ± 6.7
B	24	14 (58.33%)	8 (33.33%)	2 (8.33%)	5 (5/24, 20.83%)	84.5 ± 6.2
C	22	11 (50.00%)	3 (13.64%)	8 (36.36%)	5 (5/22, 22.73%)	74.3 ± 8.3

*Group A* anatomical reduction group, *Group B* Gotfried positive buttress reduction group, *Group C* Gotfried negative buttress reduction group, *AVN* avascular necrosis

### Statistical analysis

SPSS software was used to take the statistical analysis, normal distribution of data tested using the Shapiro–Wilk *W* test. The patient’s age, follow-up time, and Harris score were normally distributed data, exhibited homogeneity of variance. Analyses were carried out using one-way ANOVA in 3 groups. If the difference was statistically significant, then the SNK *q* test was used to compare the two groups. The *χ*2 test or Fisher exact test was used for categorical variables. *P* < 0.05 considered that the difference was statistically significant significance.

## Results

In the present study, Gotfried reduction and cannulated screw fixation were taken in all cases. Overall, 67 eligible patients were included in the analysis and followed for a median duration of 22.3 months. The average follow-up periods in the three groups were (22.4 ± 10.7), (22.1 ± 11.2), (22.8 ± 11.6) months, which showed no statistical difference (*F* = 0.053, *P* = 0.964). The median time to surgical fixation was 2 days, and the mean surgical time was 38.3 min.

There was no wound infection, fracture nonunion, and implant failure in either group. No significant differences were noted preoperatively between the three groups regarding age, time of operation, gender, and average time of follow-up (*P* > 0.05). At the last follow-up, 14 patients had avascular necrosis of the femoral head, including 4 in group A, 5 in group B, and 5 in group C.

There was no statistically significant difference in the avascular necrosis rate among the three groups (*P* = 0.156). At the last follow-up, The Harris score in patients with positive support was significantly better than those in the negative support group, close to that of the anatomical reduction group.

**Fig. 1:**
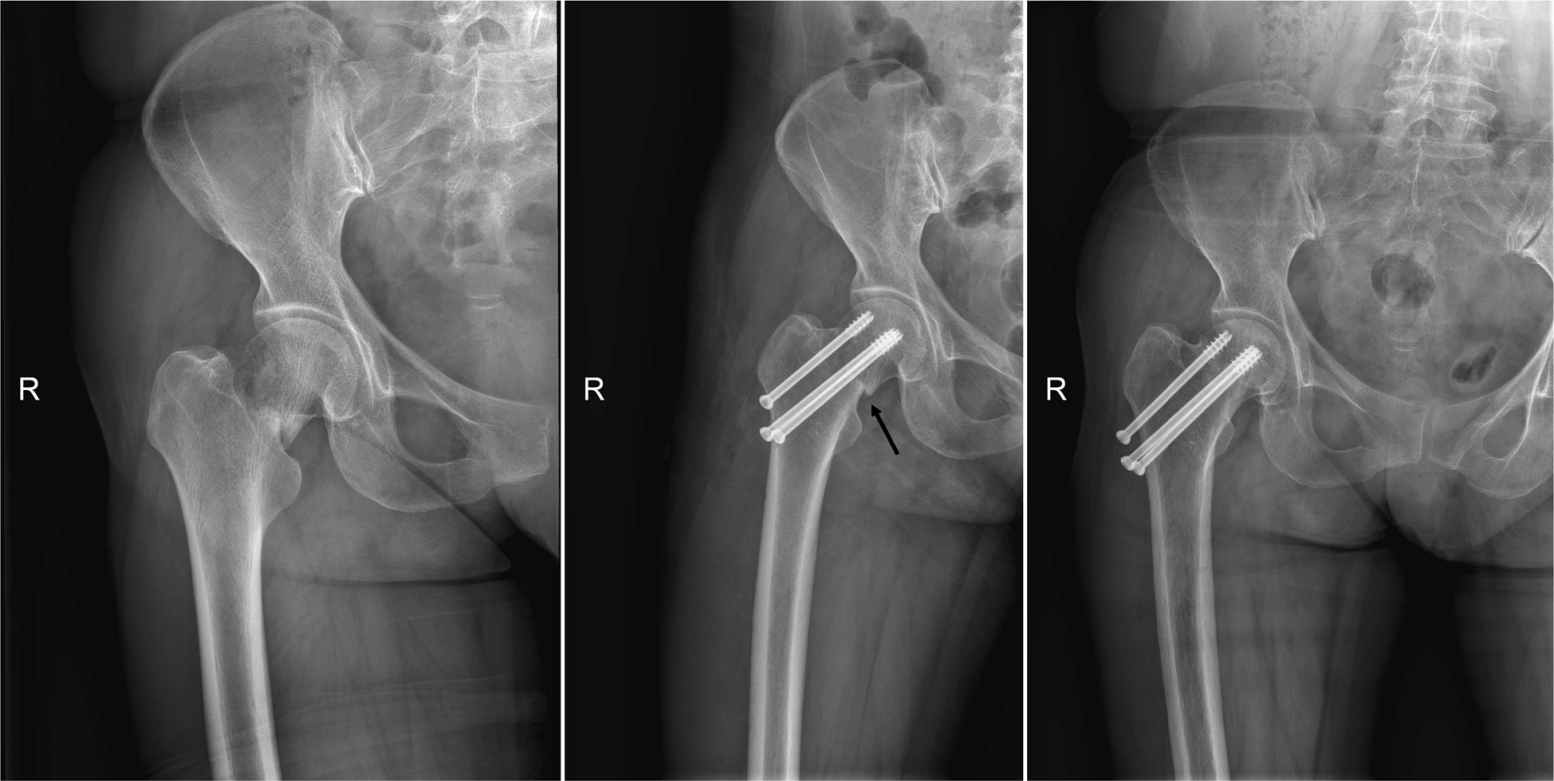
**a** Preoperative radiography. **b** Postoperative radiography and the arrow indicate negative buttress. **c** After a 2-year follow-up, the radiography shows femoral neck shortening and varus deformity

**Fig. 2:**
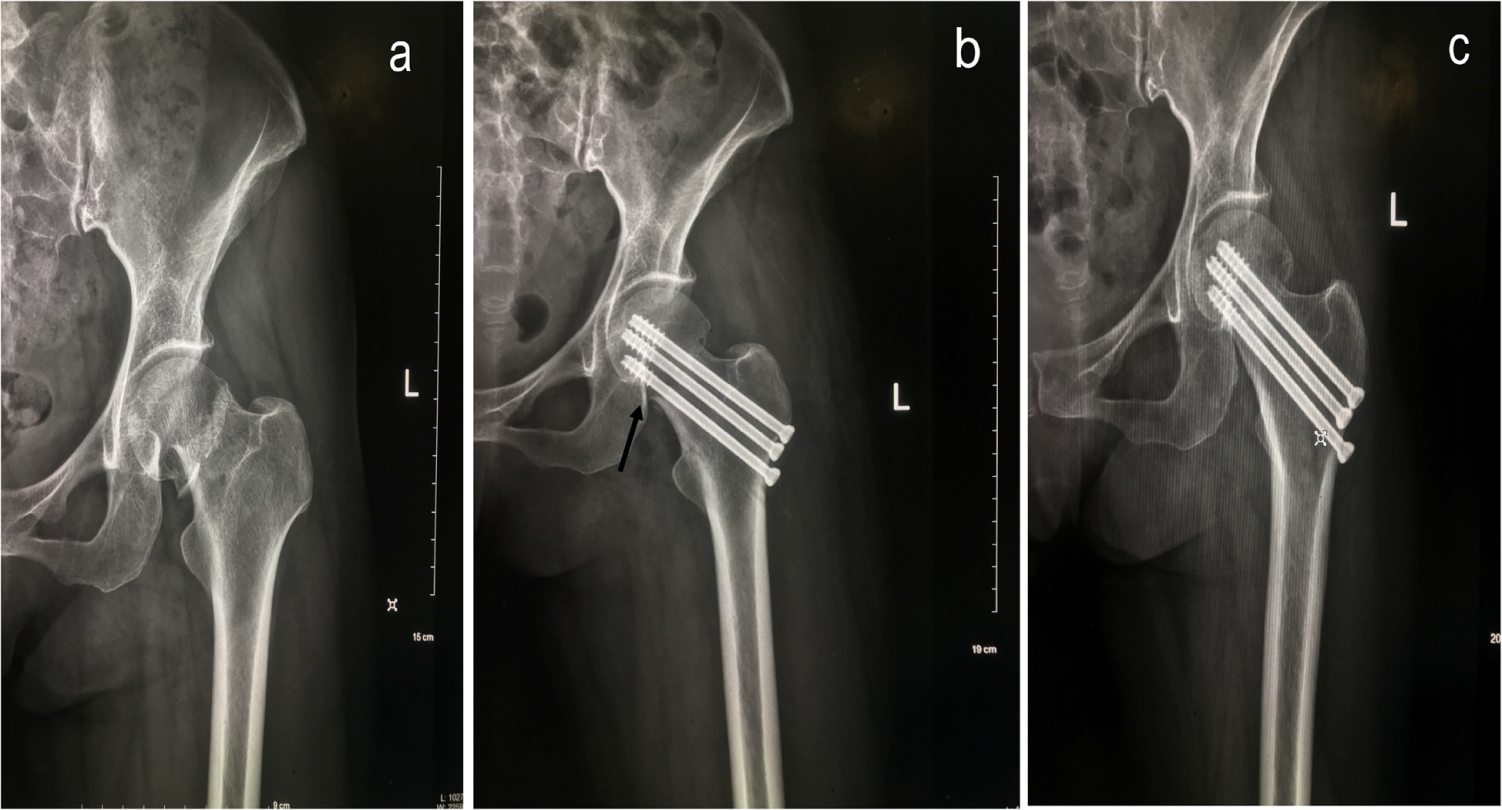
**a** Preoperative radiography. **b** Postoperative radiography and the arrow indicate positive buttress. **c** After a 2-year follow-up, radiography shows good fracture union without malunion

The incidence of femoral neck shortening in group A and group B was significantly lower than that in group C in postoperative 1 year, and the difference was statistically significant (*P* < 0.05). There was no significant difference in the short-term efficacy between Gotfried positive support group and the anatomical reduction group (Figs. 1 and 2).

## Discussion

The treatment of femoral neck fracture in young patients still faces many challenges due to adverse clinical outcomes. In a survey of 540 orthopedic surgeons, 78% of surgeons prefer to use multiple cannulated screws to treat nondisplaced femoral neck fracture. For displaced fractures, multiple screws (46%) and sliding hip screws (SHS, 49%) were first taken into consideration for the majority of orthopedic surgeons [10].

The surgical approach includes open or closed reduction with internal fixation. Ghayoumi et al. [8] found no difference in nonunion or AVN rates in their meta-analysis of the literature when comparing open versus closed reduction techniques. Most of the studies were reported higher rates of avascular necrosis, premature epiphyseal fusion, and heterotopic ossifications after open reduction. Otherwise, the closed reduction has the advantages of less trauma, less intraoperative bleeding, and shorter operation time, theoretically reduced the risk of postoperative surgical complications [8]. Consequently, closed reduction and internal fixation of fracture is the preferred method of treatment. In this study, all patients underwent Gotfried reduction (closed reduction) and cannulated screws fixation.

For younger patients (under 65 years), anatomical reduction, rigid fixation, and preservation of their hip joints are the primary treatment goals, especially to those who have an abundance of daily activities [6, 7, 9, 14]. There is a large body of literature repeatedly emphasized the importance of anatomical reduction. It has been regarded as a key to effective treatment [6, 10, 15]. Although achieving anatomical reduction is the goal of treating femoral neck fractures, it does not guarantee a good prognosis. It probably requires repeated manipulations to achieve anatomical reduction but instead appears to increase the odds of avascular necrosis and fracture nonunion [8].

The anatomical reduction was never challenged; however, Gotfried et al. believe that anatomical reduction is not strictly necessary [12]. The anatomical reduction may not promote fracture healing and reduce the occurrence of femoral head avascular necrosis [11]. In a group of young patients, there is a good reduction in postoperative radiography. However, nonunion occurred in 8%, 11.5% of avascular necrosis of the femoral head [1]. Gotfried et al. proposed the concept of “non-anatomical reduction of femoral neck fractures” and expounded the following concepts in his literature: (1) positive buttress position a displaced subcapital femoral position, anteroposterior (AP) view, in which the distal femoral neck fragment is positioned medially to the lower-medial edge of the proximal fracture fragment; (2) negative buttress position a displaced subcapital femoral position, (AP) view, in which the proximal fracture fragment (femoral neck and head) is displaced medially to the upper medial edge of the distal femoral neck fragment. (3) Negative buttress position is highly correlated with failure of reduction.

Although Gotfried reduction method was first applied to subcapitated femoral neck fractures, the concept of positive buttress is not limited to this type of fractures. This study included both subcapital, transcervical, and basicervical fracture types. The results showed that the incidence of postoperative neck shortening and Harris score of the hip joint in the positive support reduction group were significantly better than those in the negative support reduction group.

Interestingly, in this study, we found that non-anatomical reduction with Gotfried positive buttress may lead to similar clinical results with anatomic reduction. The Harris scores and neck shorting in group A (anatomical reduction) was not statistically different from group B (positive buttress reduction). Nevertheless, the first two groups are statistically better than group C (negative buttress reduction). So, the patients’ good postoperative function may be related to the advantages of positive buttress reduction in preventing neck shortening.

Parallel cannulated screws fixation is sometimes associated with a risk of femoral neck shortening. The varus and shortening of the femoral neck negatively correlate with Harris hip scores, as reported in the literature. Patients with severe shortening of the femoral neck had significantly lower short (SF-36) physical functioning scores. Shortening also resulted in a significantly lower EuroQol questionnaire (EQ 5D) index score [3, 13].

The benefits of positive buttress may come from the special anatomy of the proximal femur. The direction of forces reflects the development of the femoral neck’s trabecular lines, which increased bone mineral density to specific areas of the hip. Ward’s area, or Ward’s triangle as initially called, is the space localized at the femoral neck formed by the intersection of three trabecular bundles, namely, the principal compressive, the secondary compressive, and the tensile trabeculae, an arched structure is formed on the medial side of the femoral neck [16]. Adam’s arch plays an essential role in sustaining the stability of the femoral neck. The neck resides in an arch of compact tissue, which begins small where the globular head joins the under part of the neck, but which gradually enlarges downwards toward the lesser for the stability of the proximal femur [17]. When the positive buttress is reached, the sliding pressure of the femoral head forms an insertion. The distal of cortex supports the proximal of the medial femoral neck. The special stress of the arch structure can effectively resist the longitudinal shear force between the fractured pieces and stabilize the fracture.

The limitations of this study are similar to those of all retrospective study designs. The retrospective study and the limited follow-up may lead to significant selection bias. Furthermore, this study is limited by the small number of case series. Besides, all types of fractures were included in the study. Theoretically, vertical femoral neck fractures have a higher failure rate than other types, which may influence the final result.

We would like to recommend this procedure as an available method of closed reduction before attempting open reduction. This procedure is used to treat irreducible femoral neck fractures encountered in our trauma center as part of routine procedures, and long term follow-up for the functional outcome will be obtained to confirm the therapeutic effectiveness of this procedure further.

## Conclusion

Therefore, patients who have achieved positive support and valgus reduction may not need to pursue an anatomical reduction. Excessive pursuit of anatomical reduction will damage the blood supply and may cause avascular necrosis. We recommend that if you do not use open reduction, you need to pay attention to (1) to get an anatomical reduction as much as possible; (2) if anatomical reduction cannot be obtained, to get a positive reduction as much as possible; (3) avoid negative support.

## Data Availability

The datasets used and/or analyzed during the current study are available from the corresponding author on reasonable request.

## Abbreviations

AVN: Avascular osteonecrosis of the femoral head
HHS: Harris hip scoring
THR: Total hip replacement
AP: Anteroposterior
